# Bibliography

**Published:** 1910-06-15

**Authors:** 


### BIBLIOGRAPHY.

GUERENI'S HISTORY OF DENTISTRY. This great work is published under the auspices of the National Dental Association by Lea & Febiger, Philadelphia. It is a work of 350 pages, beautifully printed and illustrated with many fine engravings of appliances and early practitioners. We can most earnestly recommend this book to the profession as the best work on the history of dental art and practice from the earliest times to the nineteenth century. Dr. Guereni is well qualified to do the work and he has evidently spent much time and taken the greatest pains to secure accuracy in the work. The book will undoubtedly hold the first place as a reliable record of early dentistry for a long time. The Committee having the matter of publication in hand is to be congratulated and commended for the splendid style in which the dook has been made and the accuracy of its editorial supervision. It is most fascinating reading and we are certain that any one who buys the work will always highly prize it. One is struck with the many methods of practice which he is accustomed to think of as modern, which were practiced, crudely perhaps, three or four centuries ago. A Gold Crown, for instance, was made and fitted over a tooth in 1593. Extracting teeth with forceps is a much older practice than we supposed; filling teeth with gold, and other modern practices were done 300 years ago.

